# Changes in body composition across a competitive season among three division 1 female sports

**DOI:** 10.1080/15502783.2025.2550209

**Published:** 2025-08-22

**Authors:** Matthew T. Stratton, Shelley L. Holden, Austin T. Massengale, Ray Davis, Jarrett Strate-Lutzow, Kaitlyn Evenson-McMurtry

**Affiliations:** University of South Alabama, Basic and Applied Laboratory for Dietary Interventions in Exercise and Sport, Department of Health, Kinesiology & Sport, Mobile, AL, USA

**Keywords:** Athletes, body mass, competition, body composition

## Abstract

**Background:**

Collegiate athletes undergo body composition changes during their competitive seasons, yet potential differences across sports remain underexplored. This study investigated the changes in body mass (BM), fat mass (FM), body fat percentage (BF%), and fat-free mass (FFM) across a competitive season in NCAA Division I female athletes from the University of South Alabama’s soccer, volleyball, and softball teams.

**Methods:**

Fifty-seven female NCAA Division I athletes (soccer: *n* = 23; volleyball: *n* = 18; softball: *n* = 16) underwent body composition assessments via dual X-ray absorptiometry (DXA) at three timepoints across their respective competitive season: at the start of preseason, halfway through the regular season, and the week prior to their post season tournament. A repeated measures ANOVA was used to estimate changes in BM, FM, BF%, and FFM across the season between sports.

**Results:**

A significant main effect of sport was observed for BM (*p* = 0.005, η^2^_p_ = 0.179). Post hoc comparisons indicated that volleyball athletes had significantly greater BM compared to both soccer (*p* = 0.022) and softball players (*p* = 0.009), while no significant difference between soccer and softball (*p* = 0.982) was found. For FM, a significant main effect of sport was detected (*p* = 0.027, η^2^_p_ = 0.125). Post hoc pairwise comparisons revealed that volleyball players had significantly greater FM than soccer athletes (*p* = 0.027) but not significantly different from softball athletes (*p* = 0.121). Softball players also displayed significantly greater FM than soccer players (*p* = 0.046). Although no significant main effects of time were detected for BM, FM, BF%, and FFM, a significant sport × time interaction was found for BF% (*p* = 0.027, η^2^_p_ = 0.108). However, post hoc comparisons did not identify any statistically significant changes within individual sports across timepoints. Between-sport comparisons at each timepoint also failed to reach significance after adjustment for multiple comparisons (all *p* > 0.13). While the overall interaction was significant, the lack of significant pairwise differences suggests that the observed effect may reflect small, inconsistent fluctuations in BF% across time and sport rather than robust, uniform changes within any one group.

**Conclusions:**

These findings suggest potential sport-related differences in body composition profiles; however, longitudinal changes across the season appear limited. Further research with larger samples and sport-specific training or nutrition tracking may help clarify the practical relevance of these differences.

